# Supplementary material for: Mycobacterium tuberculosis FadD18 Promotes Proinflammatory Cytokine Secretion to Inhibit the Intracellular Survival of Bacillus Calmette–Guérin
Source: Cells. 2024 Jun 11;13(12):1019. doi: 10.3390/cells13121019 (PMC11201411; doi:10.3390/cells13121019)
Supplement: Supplementary file 1 [file cells-13-01019-s001.zip › Figure S1. Identification of the B2909 mutant transposon insertion site and construction of the complement strain.pdf]

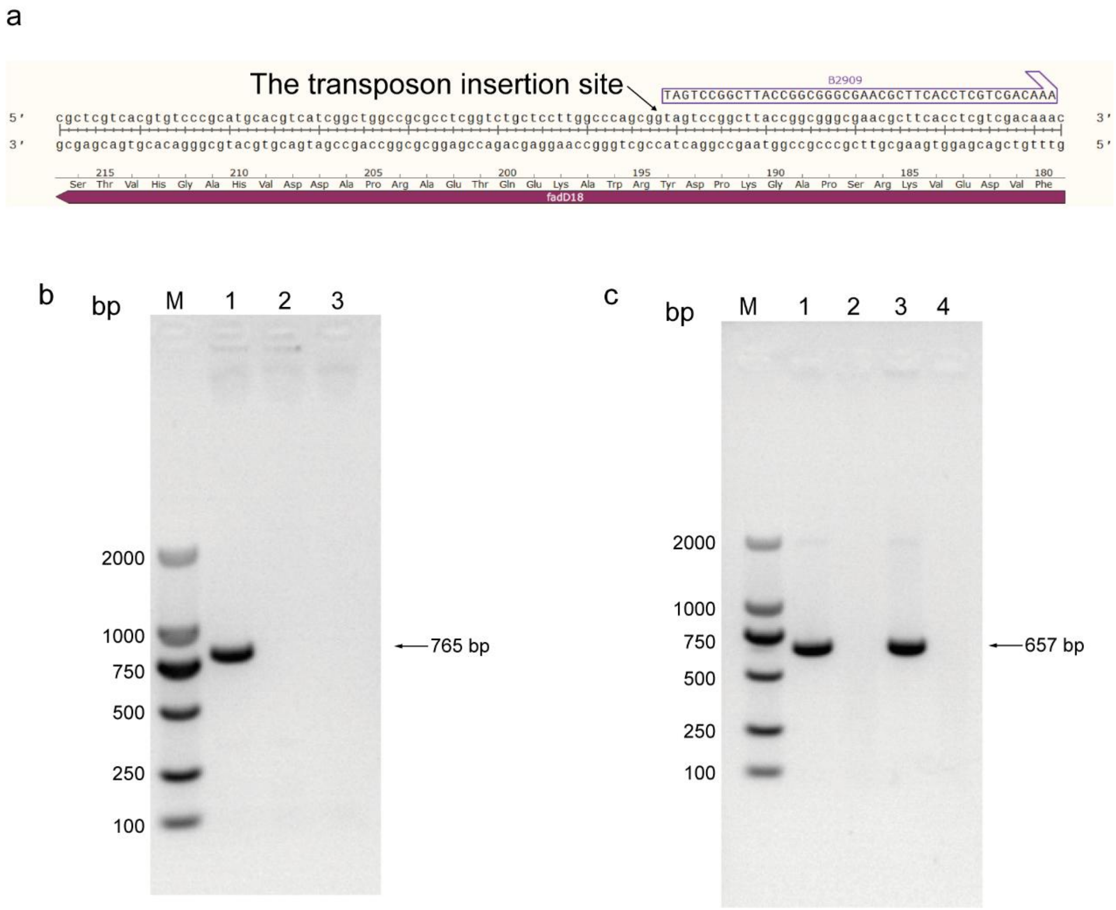

Figure S1. Identification of the B2909 mutant transposon insertion site and construction of the complement strain. (a) Insertion site map. The B2909 mutant transposon was inserted at site 581 of the gene, with a fragment size of 657 bp. (b) PCR verification of the B2909 mutant strain. Lane M: DL2000 Marker, Lane 1: B2909, Lane 2: BCG, Lane 3: Negative control. (c) Confirmation of complement strain B2909C construction via PCR. Lane M: DL2000 Marker, Lane 1: BCG, Lane 2: B2909, Lane 3: B2909C, Lane 4: Negative control.
